# Association of Pancreatitis and Biliary Affections*Read before the Post-Graduate Clinical Society, as part of a symposium on Disease of the Biliary System, March 16th, 1917.

**Published:** 1917-06

**Authors:** Chas. Gordon Heyd

**Affiliations:** Adj. Prof. Surgery, N. Y. Post-Graduate Medical School and Hospital


### BUFFALO MEDICAL JOURNAL

Yearly Volume 72 JUNE, 1917 Number 11

### ORIGINAL ARTICLES

**The right is reserved to decline papers not dealing with practical medical and surgical subjects, and such as might offend or fail to interest readers. Contributors are solely responsible for opinions, methods of expression and revision of proof.**

### Association of Pancreatitis and Biliary Affections.*

CHAS. GORDON HEYD, A. B., M. D.

Adj. Prof. Surgery, N. Y. Post-Graduate Medical School and Hospital

The pancreas represents a self-contained laboratory of physiological chemistry. Sweet (1). In the body economy it subserves two great purposes: (1) that of the digestion of food; (2) as a control or balance through its internal secretions of the glands concerned in the general functions of metabolism.

When abdominal exploration became a routine measure in clean laparotomies some very remarkable pathological associations were discovered. Not the least important of these was the association of supposed pancreatitis and disease of the biliary apparatus. It was soon discovered that in cholelithiasis there was very frequently a hard, firm pancreas, although so far as chemical examination of the stool, urine and blood was concerned there was a normal pancreatic digestion. As a corollary to these findings it was soon appreciated that a hard pancreas was not necessarily a diseased one and that normal pancreatic function could exist in a gland possessing stony hardness. Pratt (2).

In reporting the statistics of the Mayo Clinic it was found that in 2611 operations on the gall bladder, the pancreas was involved in 7.6 per cent. In 325 operations on the common duct, the pancreas was involved in 22 per cent, and in 168

*Read before the Post-Graduate Clinical Society, as part of a symposium on Disease of the Biliary System, March 16th, 1917.

operations for disease of the pancreas 81 per cent were accompanied by or due to gallstones. Mayo (3).

Mayo Robson (4) places the association of gallstones, particularly when in the common duct, with interstitial pancreatitis at about 60 per cent. Quenu and Duval (5) represented the occurrence of gallstones with chronic pancreatitis as being about equal to 50 per cent, while in 83 cases reported by Williams and Busch (6) gallstones were found in approximately 40 per cent. Egdahl (7) has found that in 105 cases of acute pancreatitis gallstones were found in 42 per cent. Erdmann (8) reported seventeen cases of acute pancreatitis with gallstones or gallbladder disease in fifteen.

It is evident that the percentage of association is somewhere around 50 per cent in the acute cases and about 65 per cent. in the chronic cases. When we consider, however, that gallstones are only found in about 75 per cent. of the diseased gallbladders that come to operation the association of biliary infection and pancreatitis is probably higher than indicated. Here, however, is a noteworthy fact, that whereas gallstones have a preponderant proportion in the female sex (four to one), pancreatitis on the other hand has a reverse proportion (three to one) in men. It is surprising then that if pancreatitis is due to infection or affections of the biliary apparatus that it does not occur in the same relative sex proportion as does cholelithiasis.

A very great impetus was given to the study of the clinical and pathological phases of pancreatitis by the post-mortem discovery of Opie (9) in 1901 of the effect of a small stone in the ampulla of Vater with the retrojection of bile into the duct of Wirsung. This discovery was given great clinical value when Flexner (11) demonstrated that pancreatitis could be produced by the injection of bile into the duct of Wirsung, and further that bile from the gallbladder was less injurious than bile from the common duct. The only essential difference between bile from the gallbladder and from the common duct was that the former is incorporated with mucin and hence the colloid secretion of the gallbladder lessens the noxious quality of bile if by chance it should be retrojected into the duct of Wirsung.

The topographical anatomy of the common duct is also of particular importance and shows that the accidental occurrence of a stone in the terminal portion of the common duct must be extremely rare. In 38 per cent. of cases the common duct passes behind the pancreas while in 62 per cent of cases it traverses the substance of the head of the pancreas. Helly (12). In 83 per cent. of cases the duct of Wirsung carries the entire pancreatic secretion, while in about 12 per cent. the duct of Santorini is the main duct. There is an anastomosis between the duct of Santorini and the duct of Wirsung in approximately 90 per cent. of cases (Opie 10), and in 54 per cent of cases the duct of Santorini may act as a substitute for the duct of Wirsung. Erdmann and Heyd (13).

The termination of the common bile duct and the duct of Wirsung is an unfortunate anatomical association, and this arrangement is subject to considerable variation. In addition, at the termination of the two ducts there is a circular band of non-striped muscle fibres known as the Sphincter of Oddi, which has great physiological importance in the ejection of bile and pancreatic secretion into the duodenum.

Letulle and Nathan Lorrier (14) describe four types. In the first type the common duct and the duct of Wirsung form an ampulla. This occurs in about 30 per cent. of cases. In the second type the duct of Wirsung joins the common duct at some little distance from its entrance into the duodenum. This occurs in about 20 per cent. of cases. In the third type the two ducts open independently into a fossa in the wall of the duodenum. This occurs in about 30 per cent. of cases. In the fourth type both ducts open independently at the apex of a caruncle on the wall of the duodenum. This occurs in about 20 per cent. of cases. Robson and Cammidge (15).

Accordingly, in only 50 per cent. of cases would the arrangement of the duct be such that a stone at the termination of the common duct could possibly cause retrojection of bile. Pancreatitis as ordinarily found in biliary disease is usually confined to the head of the pancreas, particularly to that portion of the pancreas bounded by the duct of Santorini, the duct of Wirsung and the duodenum and known as the triangle of pancreatic infection. Desjardins (16).

In the attempt to account for the etiology of pancreatitis three ideas have been advanced. First, that pancreatitis is a ductal infection from the duodenum. That infection from the duodenum is possible in some cases cannot be denied, but if pancreatitis were an infection by continuity the inflammation of the pancreas should extend throughout the ductal system. In practice such a condition is not ordinarily found. The pancreatitis, on the contrary, is of an interlobular type and one not primarily concerned with the ductal system. Moreover, the duodenum is relatively sterile (Gilbert, Dominini, Cushing 17), and neither the clinical course nor the post-mortem findings support the contention that infection by continuity of tissue is the predominant element in pancreatitis.

Mayo Robson (18) believes that many cases of catarrhal jaundice are cases of mild pancreatitis and he distinguishes two types: when the terminal portion of the common duct is involved he designates it cholangitic jaundice, and when the duct of Wirsung is involved he designates it pancreatic jaundice.

William and Busch (6) claim that the traumatism caused by the passage of a calculus results in dilatation of the Sphincter of Oddi and permits the entrance of infectious micro-organisms from the duodenum into the termination of the pancreatic duct. This is a noteworthy contribution and is probably a factor in a certain proportion of cases.

The pancreas is seldom involved in pure hemic infections so that the role of hematogenous infection is not particularly apparent, although the recent researches of Rosenow show that certain strains of streptococci have a somewhat partial selective affinity for the pancreas. Rosenow (19).

Bartels (20), Franke (21), Deaver and Pfeiffer (22) and Sweet (23) have drawn attention to the importance of the lymphatics as routes of pancreatic infection. Franke (21) was able to demonstrate that infection of the lymph gland at the sigmoid curve of the cystic duct produced lymphatic involvement of the superior pancreatic lymphnodes and further that the almost constant presence of a lymph gland between the common duct and the duodenum which would correspond to the triangle of pancreatic infection. Deaver (22).

Moreover, the intimate association between the pericolonic lymph glands and the inferior pancreatic lymph glands is worthy of note in considering the etiology of pancreatitis. The lymphatics of the appendiceal region drain into the region of the hilum of the right kidney (Franke and Kumita) 24) and there is a very distinct lymphatic connection from the hilum of the right kidney to the left suprarenal gland and the pancreas. Bartels (20).

Mayo Robson (27), Moynihan (28), Rost, and Judd (29) have drawn attention to the uniform dilatation of the common duct found in patients having a second operation after a previous cholecystectomy. In animals devoid of a gallbladder the common duct is relatively and absolutely larger than in animals possessing a gallbladder. In people with congenital absence of the gallbladder the common duct is relatively and absolutely larger than normal.

The clinical fact that cholecystectomy and cholecystostomy both cure mild affections of the pancreas was for a considerable time perplexing. The dilatation that occurs after cholecystectomy is probably sufficient to dilate the Sphincter of Oddi and so cure pancreatitis by intraduodenal drainage, while after cholecystostomy the biliary pressure is relieved by external drainage.

Archibald (25, 26) has shown that under ordinary conditions the pressure of biliary secretion plus the expulsive pressure of the gallbladder is sufficient to overcome the normal tonicity of the Sphincter of Oddi. However, the normal gallbladder is incapable of completely emptying itself, and its expulsive pressure is only exerted when the gallbladder becomes tense. Pavlov (30) demonstrated that although bile is secreted continuously it is ejected only intermittently into the duodenum. Cannon (31) drew attention to the chemical interaction of acid chyme and the duodenal mucosa bringing about a relaxation of the Sphincter of Oddi. It would appear that under certain conditions the Sphincter of Oddi might become spastic giving a condition of sphincterismus. Deaver (22).

Archibald concludes that many cases of pancreatitis are to be explained as due to chemical irritation of the pancreas from transient retrojection of bile. By means of a canula inserted into the common duct he was able to demonstrate that under the stress of a single sudden increase in biliary pressure there was a retrojection of bile into the pancreas before the Sphincter of Oddi relaxed.

Clinical proof to this contention would be suggested by a communication of Rehling (32), who in a case of acute pancreatitis incised the head of the pancreas for drainage and for some days thereafter bile came through the pancreatic drainage tube.

The fact that duodenal ulcer is so common in males, occurring in the some proportion as pancreatitis may point to a distinct association between lesions of the duodenum and the development of pancreatitis.

### CONCLUSIONS:

1. Pancreatitis is probably due to both infection and chemical irritation.
2. The very intimate lymphatic connection between the lymphatics of the pancreas and the biliary apparatus is probably a factor in many cases.
3. Gallstones have a distinct bearing upon the production of pancreatitis, being present in approximately 50 per cent. of all cases. The incidence of pancreatitis and biliary disease is probably dependent upon the anatomical variations in the terminal portion of the ducts.
4. The passage of a gallstone with injury and dilatation of the Sphincter and ampulla of Vater probably initiates infection from the duodenum.

5. Pancreatic lithiasis probably acts in like manner.
6. By reason of its peculiar anatomy infection once induced in the pancreas is probably not spontaneously cured.

BIBLIOGRAPHY.

1. Sweet, J. F.: Surgery of the Pancreas. Inter. Clinics, vol. iv., Series 25.
2. Pratt, Jos. H.: The Functional Diagnosis of Pancreatic Disease. Amer. Jour. Med. Sc., March 1912.
3. Mayo: Surg. Gynec. and Obst., Dec. 1908.
4. Robson: London Lancet, 1900, ii, 235.
5. Quenu and Duval, Rev. de Chir., Oct. 1905.
6. Williams and Busch: Trans, Assn. Amer. Phys., 1907, xxii, 304.
7. Egdahl, Johns Hopkins Hospital Bulletin, 1907, xvii, 130.
8. Erdmann, J. F.: Acute Pancreatitis, N. Y. Medical Journal, Jan. 24, 1914.
9. Opie, Eugene L.: The Pancreas and Pancreatic Disease. Tr. Congress of Amer. Phys. and Surg., vi, 1903.
10. Opie, Eugene L.: Lesions peculiar to the Pancreas and Their Clinical Aspect., Med. News, New York, May 21, 1904.
11. Flexner, Simon: The Constituent of the Bile Causing Pancreatitis and the Effect of Colloids upon its Action, Jour. Exper. Med., Jan. 1906.
12. Helly: Archiv. f. Mikroskop, Anat., 1898, lii, 773.
13. Erdmann, J. F. and Heyd, Chas. Gordon: Relief of Chronic Obstructive Jaundice by Palliative Operations, Amer. Jour. Med. Sc., clii, 2, 1916.
14. Letulle et Nathan-Lorrier: Bull. Soc., Paris, 1899.
15. Robson and Cammidge: Gallstones and Their Complications and Treatment. Oxford Univ. Press, 1909.
16. Desjardins: These de Paris, 1905.
17. Gilbert and Dominici, Cushing: Quoted by Moynihan, Abdominal Operations, Vol. i, p. 20. Saunders.
18. Mayo Robson: Pancreatic Catarrh and Interstitial Pancreatitis in Their Relation to Catarrhal Jaundice and Also to Glycosuria.
19. Rosenow, E. C.: Pathogeneses of Spontaneous and Experimental Appendicitis, Ulcer of Stomach, and Cholecystitis, Jour. Ind. State Med. Assn., 1915, viii.
20. Bartels: Arch. f. Anat. u. Phys., Anat, Abtl., 1904, 299; 1906, 250; 1907, 267.
21. Franke: Deutsch. Zeitsch. f. Chir. September 1911.
22. Deaver, John B. and Pfeiffer, Damon; Pancreatic Lymphangitis, Amer. Jour. Med. Sc., April 1912.
22. Deaver, John B. and Pfeiffer, Damon; Pancreatic and

Peripancreatic Lymphangitis, Trans. Amer. Surg. Assn., 1913.
22. Deaver, John B.: Acute and Chronic Pancreatitis, Arch. Diagnosis, Jan. 1912.
22. Deaver, John B. and Pfeiffer, Damon: Chronic Pancreatitis, Ann. Surg., June 1914.
23. Sweet, J. F. and Stewart, L. F.: The Ascending Infection of the Kidneys. Surg. Gynec. and Obst., April, xviii, 4.
24. Kumita: Lymphgefasse de Mieren-und Nabernierenkapsel. Arch. f. Anat. u. Physiol., Anat. Abt., 1909, 49.
25. Archibald, Edward: Ideas concerning the Causation of Some Cases of Pancreatitis, Canad. Jour. Med. and Surg., 1912.
26. Archibald, Edward: A new Factor in the Causation of Pancreatitis, 17th International Congress of Medicine. London, Aug. 1913.
27. Robson and Cammidge: The Pancreas, Its Surgery and Pathology. Saunders.
28. Moynihan, B. A.: Gallstones. Saunders.
29. Judd, E. S.: Cholecystitis: Changes Produced by the Removal of the Gallbladder. Boston Med. and Surg. Jour., clxxiv, 23, 1916.
30. Pavlov, I. P. and Thompson, W. H.: The Work of the Digestive Glands. C. Griffin and Co.
31. Cannon: Mechanical Factors of Digestion.
32. Rehling: Personal Communication.

---

**Poliomyelitis.** Horace Greeley, Brooklyn, Boston M. & S. Jour., April 12, has found that Rosenow's bacillus grows freely on milk at 70° F. and upward, and he believes that this has a double bearing on the etiology. Of 60,000 cases reported in epidemics, less than 30 were among breast-fed children. Moreover, the incidence corresponds closely with temperatures favoring the growth of the bacillus on milk. For instance, in N. Y. City, the mean temperature was 59.3° with 29 cases for May; 64.2°, 756 cases for June; 73.8°, 3863 cases for July; 73.6°, 3306 cases for Aug.; 66°, 780 cases for Sept.; 57.2°, 193 cases for Oct. This article is the most definite one on etiology that we have seen, and the contentions should be thoroughly examined. It may be pointed out, however, that the influence of temperature on incidence may depend on the action on carriers rather than germs in the direct sense—as in regard to yellow fever, whose germ was long regarded as proved to be killed by cold, whereas it was really the stegomyia that was frozen.

